# Perspectives of international experts and the Danish citizens on the ‘relevant knowledge’ that citizens need for making informed choices about participation in cancer screening: Qualitative study

**DOI:** 10.1016/j.pec.2024.108479

**Published:** 2024-10-24

**Authors:** Rikke Nicoline Stokholm, Pia Kirkegaard, Mette Bach Larsen, Henrik Hein Lauridsen, Dawn Stacey, Diane M. Harper, Karen Sepucha, Kirsten McCaffery, Maren Reder, Michael Pignone, Mirjam Fransen, Robert J. Volk, Yvonne Wengström, Adrian Edwards, Berit Andersen

**Affiliations:** a University Research Clinic for Cancer Screening, Department of Public Health Programmes, Randers Regional Hospital, Randers, Denmark; b Department of Clinical Medicine, Aarhus University, Denmark; c Research unit, Horsens Regional Hospital, Denmark; d Department of Sports Science and Clinical Biomechanics, University of Southern Denmark, Denmark; e Ottawa Hospital Research Institute, University of Ottawa, Canada; f University of Michigan, Ann Arbor, MI, USA; g Massachusetts General Hospital, Harvard Medical School, Boston, MA, USA; h Sydney Health Literacy Lab, School of Public Health, Faculty of Medicine and Health, The University of Sydney, Sydney, NSW, Australia; i Institute of Psychology, University of Hildesheim, Hildesheim, Germany; j Duke University, Department of Medicine, Durham, USA; k Department of Public and Occupational Health, Amsterdam University Medical Center, University of Amsterdam, Amsterdam, the Netherlands; l Amsterdam Public Health Research Institute, Quality of Care and Digital Health, Amsterdam, the Netherlands; m Department of Behavior and Health, Centre for Prevention, Lifestyle and Health, National Institute for Public Health and the Environment, Bilthoven, the Netherlands; n Department of Health Services Research, The University of Texas MD Anderson Cancer Center, Houston, TX, USA; o NVS, Karolinska Institutet and Karolinska Comprehensive Cancer Center, Karolinska University Hospital, Stockholm, Sweden; p PRIME Centre Wales, Division of Population Medicine, School of Medicine, Cardiff University, UK

**Keywords:** Cancer screening, Knowledge, Decision-making, Informed choice, Breast cancer screening, Colorectal cancer screening, Cervical cancer screening

## Abstract

**Objectives:**

This study aimed to investigate the perspectives of international experts and Danish citizens on relevant knowledge about population-based breast, colorectal and cervical cancer screening.

**Methods:**

This was a qualitative interview study with focus group interviews with experts and Danish citizens eligible for breast, colorectal and/or cervical cancer screening. Data were collected using semi-structured interview guides, audio-recorded and transcribed verbatim. A thematic analysis was conducted.

**Results:**

Participants were nine international experts from Germany, Canada, the USA, Sweden, the Netherlands and Australia, and 54 citizens from Denmark. Most citizens had ‘adequate’ or ‘problematic’ levels of health literacy. Themes that experts and citizens agreed on were: knowledge about the disease and symptoms, practical information about screening, benefits of screening, the option of non-participation and the importance of having numeric information of possible screening outcomes. Experts agreed on the importance of knowledge about the harms of screening, but only a minority of citizens considered this important.

**Conclusions:**

The experts and citizens disagreed on the relevance of knowledge about harms of screening and agreed on other relevant knowledge.

**Practice implications:**

What experts and citizens find important may not align when making informed decisions. Therefore, experts and citizens needs to be involved when developing questionnaires.

## 1. Introduction

Population-based cancer screening programmes advocate for citizens to make an informed choice about whether to participate in cancer screening. An informed choice is one based on relevant knowledge and demonstrates consistency between the decision-maker’s values and actual behaviour [1]. Thus, obtaining relevant knowledge is central, but the definition of relevant knowledge is not straightforward.

Few studies have investigated citizens’ views on relevant knowledge about cancer screening to make an informed choice [2,3]. A study by Jepson et al. found that people in the United Kingdom invited to screening for breast, colorectal and/or cervical cancer screening wanted information on the risk factors and symptoms related to the disease being screened for, the screening process and consequences [2]. Another study showed that Danish men and women with lower educational attainment preferred a clear recommendation about colorectal cancer screening participation from the health authorities, but some citizens also wanted comprehensive information before making the decision [3]. Further, interviews with German and Turkish women about informed choice in mammography screening indicated that being informed was not a priority due to a lack of interest among the women and it was not seen as helpful for the decision [4].

A prerequisite for measuring and comparing knowledge is consensus about what relevant knowledge is and, preferably, a consensus-driven validated questionnaire. Many studies measured knowledge about cancer screening. However, a systematic review of the validity of instruments to measure knowledge about cancer screening using the COnsensus-based Standards for selection of health Measurement INstruments (COSMIN) guidelines indicated a lack of attention to this conceptualisation of knowledge about cancer screening with inadequate development and content validation [5]. COSMIN considers conceptualisation and content validity as the most important stages for instrument development as they are essential for subsequent item generation. It is usually based on thorough qualitative methods with relevant experts [6].

Thus, we undertook a qualitative study of the concept of relevant knowledge about cancer screening that people need to make an informed choice, investigating the perspectives of both international scientific experts and Danish citizens.

## 2. Material and methods

### 2.1. Design

This study was a qualitative interview study related to the first criterion of the COSMIN guideline for the development of measurement instruments.

### 2.2. Cancer screening programmes in Denmark

Denmark has three organised cancer screening programmes:

- Citizens aged 50–74 years are offered biennial screening for colorectal cancer with Faecal Immunochemical Testing (FIT). Citizens receive an invitation by mail together with a self-sampling kit, sampling instructions, an informational pamphlet and a prepaid return enveloped to return the sample to the laboratory.
- Women aged 50–69 years are offered biennial screening for breast cancer. Women receive an invitation for screening by digital mail with a pre-booked appointment for a screening mammography at a screening centre.
- Women aged 23–64 years are offered cervical cancer screening every 3 or 5 years, depending on their age and screening modality (Human Papillomavirus (HPV) or cytology). Women receive an invitation by digital mail to call their general practitioner for a gynaecological examination. Self-sampling for HPV-testing is currently being implemented for non-participants.

### 2.3. Participants

The study participants were international scientific experts and Danish citizens. The experts were shortlisted based on publications within the field and purposively sampled to reflect the Organisation for Economic Co-operation and Development (OECD) countries in which cancer screening methods and evaluation may be comparable. The criteria for experts were that they had published within the field of informed decision-making, shared decision-making, development or evaluation of instruments or decision aids, all preferably related to breast, colorectal and/or cervical cancer screening. The experts were approached through email.

The Danish citizens had to be eligible for at least one of the three Danish cancer screening programmes. Recruitment of citizens was done via an external professional recruitment company, Norstat, which recruited the citizens through a panel consisting of Danish citizens who had voluntarily chosen to participate. The citizens were first screened against the selection criteria and afterwards they were contacted by phone. The citizens were recruited between 23 October and 22 November 2022. The authors instructed Norstat before the initiation of recruitment.

### 2.4. Data collection

**Fig. 1.**
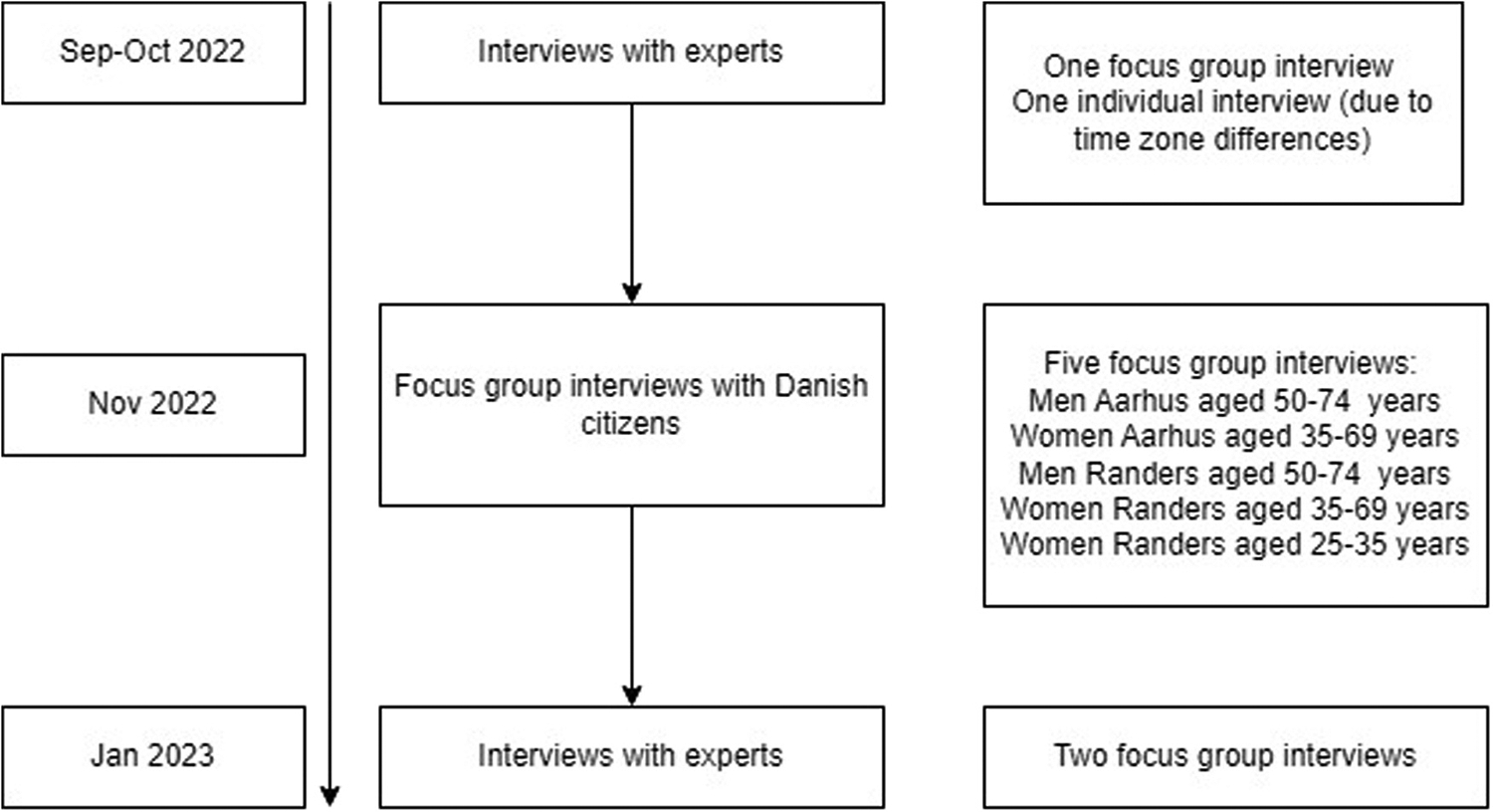
Timeline for data collection.

Data were collected through three rounds of interviews. The first round of interviews consisted of one focus group interview (FGI) with the experts, and due to time zone differences, one individual interview with an expert. The second round of interviews consisted of five FGIs with Danish citizens and the third round of interviews consisted of two FGIs with the experts (Fig. 1). All interviews with the experts were conducted online and undertaken in English, and the FGIs with Danish citizens were undertaken in Danish.

The aim of the first round of interviews with the experts was to explore perspectives on relevant knowledge about cancer screening. These began with a presentation of the project and the aim of the interview.

For the second round of interviews sixty Danish citizens were invited to take part in one of five FGI in either Aarhus or Randers (Fig. 1). Aarhus is the second biggest city in Denmark, while Randers is a medium-sized city. These cities were selected to recruit citizens from two different areas of the Central Denmark Region. In both Aarhus and Randers, we had one FGI for men aged 50–74 years (eligible for colorectal cancer screening) and one for women aged 35–69 years (eligible for colorectal, breast and/or cervical cancer screening). In Randers, we supplemented with one FGI for women aged 25–35 (eligible for cervical cancer screening). When the citizens attended the FGI, they were first asked to complete a short questionnaire about their sex, age, highest completed education, participation in screening for the aforementioned screening programmes and to fill out the European Health Literacy Survey Questionnaire (HLS-EU-Q16) [8,9]. To ensure that all participants understood the questions, the health literacy (HL) questions were read aloud. The FGI began with a short presentation of the aim of the study. Subsequently, the citizens were asked questions exploring their perspectives on the knowledge they felt they needed to make an informed decision about participation in cancer screening. They were presented with some early findings from the interviews with the experts and existing information materials already used in the Danish screening programmes, then asked to discuss whether the presented information materials were relevant or adequate for making an informed decision.

The aim of the third round of interviews, which was with the experts, was to explore differences and similarities between the experts´ and citizen´ perspectives, and they began with a presentation on the results of the analyses of the first interview and the results of the FGIs with Danish citizens. Further, the aim was to reach consensus about what defines relevant knowledge about cancer screening and define the elements that are important to include in an instrument measuring knowledge about cancer screening.

For all rounds of interviews data collection was based on semi-structured interview guides (Appendix A). The interview guide used for the Danish citizens was informed by the first interviews with the experts, and the interview guide for the third round of interviews was informed by the first interviews with the experts and the FGI with Danish citizens. Information power was assessed during data collection so data were obtained until there were enough perspectives to describe some patterns and satisfactorily achieve the aim of the study [7].

For the first round of interviews, the first FGI with the experts was undertaken by co-author AE and the remaining interviews with the experts and Danish citizens were undertaken by the first author RNS and supervised by the second author PK. All researchers are experienced in conducting interviews.

### 2.5. Data-management

All interviews were audio-recorded and transcribed verbatim by the first author and an assistant. Demographic information about the citizens, their screening status and HL were descriptively analysed using STATA. The citizens’ HL was categorised as ‘inadequate’ (0–8 points), ‘problematic’ (9–12 points) and ‘adequate’ (13–16 points). Missing items were scored as 0, and citizens were excluded from the analysis if more than two items were missing [9–11].

### 2.6. Data analysis

Thematic analysis was conducted, inspired by Braun and Clarke [12]. The analysis was conducted using Nvivo. The analyses were conducted similarly for both the experts and the citizens, but each dataset was analysed separately. First, all the transcriptions were read systematically, and initial ideas were noted by the first author (RNS). An initial coding was conducted, and codes were collated into potential themes by RNS. The themes were reviewed and a thematic map for analysis was generated by RNS and PK. RNS and PK initially discussed the codes and potential themes. The potential themes were then presented and discussed with MBL, BA and AE. The reviewed themes, thematic map and any discrepancies in the coding were discussed and resolved. Each theme was specified and refined. After the data were analysed separately, we mapped the similarities and differences of the experts’ and citizens’ perspectives.

### 2.7. Trustworthiness

To strengthen the credibility of the data, RNS and PK independently analysed the transcripts, and the initial coding and potential themes were reviewed by PK. The potential themes were discussed with MBL, BA and AE, and the final themes were reviewed by the other co-authors. To ensure transferability and dependability, the setting and research procedure were thoroughly described and documented [13].

### 2.8. Research ethics

Research projects based on interview data do not require formal ethical approval in accordance with Danish legislation. The project is listed in the record of processing activities for research projects in the Central Denmark Region (R. No.: 1–16–02–50–22).

For the interviews with the experts, oral and written consent were obtained before the interviews and for the FGI with citizens it was obtained on the day of interview. All participants were informed that their data would be anonymised, and they were allowed to withdraw consent before publishing. The experts were invited at the outset to participate in a publication arising from the interviews.

## 3. Results

### 3.1. Characteristics of the scientific experts and the Danish citizens

For the first interviews with the experts seven experts participated: one from Germany, one from Canada, four experts from the USA and one from Australia. For the second interviews with the experts, nine experts participated. These were the same experts from Germany, Canada, the USA and Australia, plus two additional experts from Sweden and the Netherlands. All the experts have conducted research within the field of decision-making and within the field of cancer and/or screening. The experts were both men and women, the age range was 36–63 years and the years of research experience within the field was 10–33 years (Table 1).

The average length of the interviews with the experts was 1 h and 30 min.

In total 54 of 60 invited citizens participated (90 %). The age of the women ranged from 25 to 69 years and 51 to 74 years for the men. Regarding screening participation, 91.67 %, 91.67 % and 92.59 % participated in the breast, colorectal, and cervical cancer screening programs, respectively. Regarding levels of HL, 39.22 % were in the category ‘adequate HL’ while 50.98 % were in the category ‘problematic HL’ (Table 2).

The average length of the interviews with the citizens was 1 h and 52 min.

### 3.2. Perspectives on relevant knowledge about cancer screening

The following five themes were identified through analysis: (1) Disease and symptoms, (2) Screening composition, (3) Benefits and harms, (4) Numeric information and (5) The option of non-participation. These are summarised narratively, and quotations presented in Table 3.

#### 3.2.1. Theme 1: Disease and symptoms

According to the experts, it is important that citizens possess knowledge about the disease and symptoms for which they undergo screening. The experts felt that without adequate comprehension of the disease and its symptoms, citizens were unable to engage in informed decision-making.

Several citizens mentioned that it is important for their decision-making to have knowledge about the disease for which they are being screened and emphasised the significance of being informed about symptoms, noting that one may not necessarily experience these symptoms. For some citizens, having information about the disease and symptoms was not essential for making an informed decision about participation in cancer screening.

#### 3.2.2. Theme 2: Screening composition

The experts considered knowledge about the screening composition essential, including the aim of screening, screening pathway and practicalities, the target group of screening and that screening is a repeated process.

The experts described that knowledge about screening procedures, follow-up tests if indicated and understanding that an abnormal screening result does not necessarily mean that the citizen has cancer were all important. Important information about the procedures was understood to vary across the screening programmes. The experts mentioned that citizens participating in colorectal cancer screening need information about the procedure of the screening test and follow-up test because it requires more preparation if they have done a FIT and need to come back for a colonoscopy.

The citizens felt that information about the screening process was essential to make an informed decision about participation in cancer screening. In the decision-making process, it is paramount to know what to expect during testing. This includes procedures, participation guidelines, post-screening events and how, when and where to get the results of the screening tests.

Some citizens emphasised the importance of being informed about potential screening outcomes. They felt it essential to be prepared for what might follow screening. Additionally, they felt it essential to know that a positive screening result does not necessarily mean you have cancer. Furthermore, it was considered crucial for some citizens to beinformed about the target population of cancer screening, especially knowing at which age they would receive the final screening invitation.

#### 3.2.3. Theme 3: Benefits and harms

The experts felt it important that citizens understand that there are both benefits and harms of screening to make an informed choice, even if such information (of harms) may not be wanted by citizens. Isolated knowledge about the benefits would be inadequate and the information should be about both benefits and harms. The experts mentioned that detailed knowledge about the harms might not be necessary.

Experts expressed that adequate knowledge about harms of screening means that citizens need to know about risks, false positive/false negative test results, over-diagnosis and over-treatment. From the experts’ perspectives, it was more important that the citizens understand the concept of these harms versus their magnitudes.

The citizens felt it crucial to be informed about the benefits of screening and mentioned that it is essential for them to understand the individual benefits associated with screening.

A minority of citizens mentioned it was crucial and essential for them to receive information about both the benefits and harms of screening. However, most citizens found it was challenging to understand concepts such as false positives, false negatives, over-diagnosis and overtreatment, and some citizens questioned why they were invited for screening while simultaneously being informed that the answer of the test could be subject to uncertainties.

#### 3.2.4. Theme 4: Numeric information

The experts felt some numeric information about potential outcomes was essential for making an informed choice about participation in cancer screening.

The experts also felt it was essential for citizens to understand the likely outcomes that could follow participation in the screening programme and that this could be based on a ‘ballpark’ sense of the numerical information rather than detailed or highly accurate information.

Several citizens identified the importance of numeric information about screening outcomes for informed decision-making. Most citizens also expressed that numeric information contributed to making their decision more ‘fact-based’ and that the numeric information should be ‘simple’. Most citizens felt numeric information was useful for understanding both the risk of getting cancer and the probability of avoiding death from cancer through screening. However, some citizens felt that numeric information was not relevant for their decision-making process.

#### 3.2.5. Theme 5: Option of non-participation

The experts felt it was essential for citizens to have knowledge about the possible options presented in a balanced way. This includes the potential consequences of choosing not to participate.

The experts discussed that it could be different between screening programmes and countries regarding presenting information about the option of non-participation. In some of the represented countries, the decision regarding participating in colorectal cancer screening focuses more on selecting the appropriate screening test, while breast and cervical cancer screening is a decision about whether to participate.

Most of the citizens mentioned the option of non-participation as essential in the decision-making process. Some citizens felt that this was just as important as information about participation itself.

On the other hand, some citizens mentioned that they already knew that non-participation was an option when they received the invitation. Their reasoning was that the word ‘invitation’ indicates that it is an offer and voluntary. Furthermore, most citizens expressed that it is more essential in the decision-making process to know that they have the option to participate later if they decided not to participate in some of the screening rounds.

Table 4 summarises the differences and the similarities of the perspectives described (Table 4).

## 4. Discussion and conclusion

### 4.1. Discussion

International scientific experts and Danish citizens agreed that the concept of relevant knowledge should include knowing facts about the disease and symptoms, screening composition and the option of non-participation when making an informed decision about participating in cancer screening. Furthermore, they agreed that simple numeric information would be helpful to make a more fact-based decision. While the experts indicated that it was essential to have knowledge about both the benefits and harms of screening, most citizens stated that knowledge about harms was less important for them.

The main strength of our study is that it represents both the citizens’ and experts’ perspectives on relevant knowledge about cancer screening, which gave us the opportunity to explore similarities and differences. The experts were from different countries in Europe, the USA, Canada and Australia, which is a strength for the transferability of the experts’ perspectives. To collect as many perspectives as possible, but also mitigate the inconvenience of time difference, we allowed one expert to participate in an individual interview. The main limitations were that most of the citizens had participated in screening, so results might have differed if more citizens had not participated in screening. Further, a limitation for the transferability of the results was that we only included Danish citizens, and perspectives on relevant knowledge about cancer screening in this study requires corroboration in other countries, as findings may differ between countries. In particular, the results regarding screening composition may vary by context (e.g., screening programmes differ between countries). Furthermore, we only included experts from the OECD countries, which may limit transferability to developing countries as perspectives may be different in lower income countries. Finally, we have only investigated perspectives on breast, colorectal and cervical cancer screening, as these are the most commonly implemented programmes in the OECD countries and the only population-based cancer screening programmes offered in Denmark, but results may not be transferable to other cancer screening programmes.

To our knowledge, this is the first study investigating and seeking consensus from both citizens’ and experts’ perspectives on relevant knowledge about cancer screening before someone decides on participation. Earlier studies of citizens’ perspectives generally agree with our findings on what constitutes relevant knowledge about cancer screening. These studies found that citizens want knowledge about disease and symptoms, the procedure, benefits of screening, simple numeric knowledge, and information about the incidence and mortality, risks and limitations [2,3]. Further, a study by Woudstra et al. found that experts mentioned that it was important to weigh pros and cons, while the individuals mentioned participation as self-evident and many did not focus on the consequences of screening [14].

In 2005, the International Patient Decision Aids Standards (IPDAS) published criteria for developing patient decision aids. These criteria indicate it is important for a decision aid for screening to include both benefits and harms by describing what the test is designed to measure, the chances of true positive/negative and false positive/negative test results, the follow-up process, the chances of diagnosis with or without screening and detection or treatment that would never have caused problems if someone were not screened [15,16]. This list of criteria is consistent with the experts’ perspectives in our study. However, most of the citizens mentioned that knowledge about the harms was not important for their decision-making. A potential explanation could be that citizens prefer a clear recommendation from the health authorities about participation without considering potential harms, despite the fact that all medical tests and procedures require an explanation about harms [3]. A recent qualitative study by Fransen et al. found that information about potential harms was not considered relevant for the decision-making process by Dutch breast cancer screening invitees [17]. The study indicated that the focus on the benefits of screening can be explained by a lack of knowledge about the harms. This may also explain why most citizens in our study mentioned that knowledge about the harms was not important for their decision-making process. Other studies have indicated a lack of awareness and understanding of over-diagnosis or over-detection [18,19]. Further, a review indicated that several studies conclude that harms of population-based screening is challenging for citizens to understand and there is little guidance on how to communicate the harms of screening [20].

Additionally, a study by Kolthoff et al. indicated that the invitation for cervical cancer screening in several Scandinavian and Englishspeaking countries emphasised the benefits over the harms of screening and generally providing poor and biased information [21]. Both the experts in our study and the existing literature emphasise that an informed choice requires information about both benefits and harms of cancer screening [22,23], even though it potentially leads to fewer participants in cancer screening [24]. Our findings indicate that the citizens felt it was hard to understand the harms of screening. This emphasises the importance of thoroughly considering the strategy of communicating harms of screening to citizens so they can understand and use them when making decisions about screening participation. Using an effective communication strategy is essential when promoting informed decisions about cancer screening.

### 4.2. Conclusion

In conclusion, this study showed that international scientific experts and Danish citizens largely agreed on what constitutes relevant knowledge about cancer screening. However, a majority of the citizens did not find knowledge about the harms of screening important for their decision-making process. The results of this study should be taken into account when developing information materials for cancer screening and questionnaires to measure knowledge about cancer screening.

### 4.3. Practice implications

This study addresses COSMIN’s requirements for the development of patient-reported outcome measures (PROM) [6]. When developing items, COSMIN recommends involving experts, which in this case are both scientific experts within the field and Danish citizens. The scientific experts within the field have extensive knowledge from their research about what citizens must know about cancer screening to make an informed choice. At the same time, it is the citizens who must make the decision about whether or not to participate in cancer screening, thus their perspectives on what defines relevant knowledge in the decision-making process are also essential to consider. According to COSMIN, the best way to involve experts to gain knowledge about the concept is through FGI [6]. Following the COSMIN guideline for development of measurement instruments, the themes of what defines the concept of relevant knowledge about cancer screening that we identified in this study can be used to determine relevant items for an instrument for the measurement of knowledge about breast, colorectal and cervical cancer screening. This would provide a sound basis for instrument development and assessment of measurement properties, including content validity, structural validity, internal consistency, reliability and construct validity. Having a fully validated instrument that meets the COSMIN criteria is essential for the field of cancer screening to assess the effectiveness of interventions to enhance informed decision-making [5,25,26].

## Figures and Tables

**Table 1:** Characteristics of the experts.

All experts, N = 9	n (%)
**Sex**	
Men	2 (22.22)
Women	7 (77.78)
Age, years, *mean (range)*	54.11 (36 –63)
Research experience within the field, years, *mean (range)*	23.89 (10 –33)

**Table 2:** Characteristics of the citizens.

All citizens, N = 54	n (%)
Sex, women	31 (57.41)
**Age, years**	
Men, *mean (range)*	62.65 (51 –74)
Women, *mean (range)*	45.55 (25 –69)
**Highest education**	
Primary school	1 (1.85)
High school	2 (3.70)
Vocational education	21 (38.89)
Short higher education	3 (5.56)
Medium higher education	22 (40.74)
Long higher education	5 (9.26)
**Screening status**	
Breast cancer (N = 12 eligible for breast cancer screening)	
Yes	11 (91.67)
No	1 (8.33)
Colorectal cancer (N = 36 eligible for colorectal cancer screening)	
Yes	33 (91.67)
No	3 (8.33)
Cervical cancer (N = 27 eligible for cervical cancer screening)	
Yes	25 (92.59)
No	2 (7.41)
**Level of HL** ^a,b^	
Adequate	20 (39.22)
Problematic	26 (50.98)
Inadequate	5 (9.80)

a HL = Health Literacy,

b Citizens excluded from the analysis due to missing data= 3

**Table 3:** Quotations.

Theme	Scientific experts	Citizens
**Disease and symptoms**	’*So, so – we see that all the time in like cancer treatment questions that people don’t have a really good understanding of the disease, the cause, symptoms and so on, so everything else doesn’t matter, because they really can’t participate in informed decision-making or shared decision-making, because they lack this foundational understanding of their disease.’ (Expert within colorectal cancer screening and decision-making)*	*’Information about what this disease is, and that it is possible to have it without experiencing any symptoms.’ (Woman, 35 –69 years)* *’Something about the disease. What kind of disease it is, I know what cancer is, but what does it mean to have it (cancer).’ (Man, 50 –74 years)* *’For me it is not crucial to read about the symptoms, because if I had some symptoms like that, I would go to my general practitioner’. (Woman, 25 –35 years)*
**Screening composition**	*’[…] what is going to happen and if the test is positive or the test is negative what’s likely to happen and starting from there.’(Expert within colorectal cancer screening and decision-making)* *’[…] and I do think some of the basics - like what it is and why are we doing it and what can you expect.’ (Expert within patient decision aids, shared decision making and colorectal cancer screening)*	*’Why do we do it. What can happen if I don’t do it. I think a lot of people need to know that it is not something we just do for fun, but what the importance of it is.’ (Woman, 25 –35 years)* *’I remember it was breast examination, I remember I thought; ’How much clothing do I need to take off, and are we standing in a long queue?’ […]. I wish I had known that we entered one at a time.’ (Woman, 35 –69 years)* *’How it should be done.’ (Man, 50 –74 years)* *’[…]. Now that I have waited for my answer in 3 –5 weeks, what can I expect to be told and what should happen when I read the response’. (Woman, 35 –69 years)* *’[…]. You are told that either you are cleared or otherwise you have to be checked a bit more. That is helpful to know.’ (Man, 50 –74 years)*
**Benefits and harms**	*’[…] obviously people need to understand what the benefits of screening are and they need to understand what the harms of screening are and particularly those harms vary depending on screening procedure.’ (Expert within patient communication, shared decision-making, health literacy and cancer screening)* *’[…] Just because that people think it’s that they don’t want the information, doesn’t mean that we shouldn’t give it to them, right, that’s – I mean if you offer screening and you want informed consent, you will have to inform them about over-diagnosis. It’s a simple fact.’ (Expert within informed decision-making in breast cancer screening)* *’I think it is most important that you understand that there is such a thing as a false positive before you understand the magnitude of false positives.’ (Expert within colorectal cancer screening and decision-making)*	*’What positive outcome do I get from doing this? What do I gain from it? Because that’s often the consideration. Now I’m about to do something, invest some time in it, what benefit does it bring? It’s also what contributes to weighing up the pros and cons, and what helps in making my decision, what significance does it have, does it really make a difference? […]. Can it help, I mean.’ (Woman, 35 –69 years)* *’[…]. I mean, the only rational thing to do is to lay out all the data, […] otherwise it becomes very narrow-sighted. […]. If you present one thing, you should also present the other […].’ (Woman 35 –69 years)* *’In terms of deciding whether or not to participate, I don’t think it is important. I find it (the harms of screening) confusing. I am invited, and then I am told why I should not accept the invitation. It makes no sense to me.’ (Woman, 35 –69 years).*
**Numeric information**	*’So it is important [that] decision aids include information about numeric risk of those benefits or the probabilities of those benefits and harms. And it is important that people understand them.’ (Expert within patient communication, shared decision-making, health literacy and cancer screening)* *’I have not found that getting perfect numeric estimates is necessary but people have to understand what are the likely things that could happen throughout the process and what are the hassles that you have to go through, and that’s the very core knowledge.’ (Expert within colorectal cancer screening and decision-making)* *’But it needs to be a sort of ballpark or at least conceptually that they know that, you know, it’s a kind of handful of women whose deaths are avoided and it is in the hundreds who will have a false positive and have to have a biopsy. So that’s where we kind of landed that people needed to have a kind of ballpark sense of the benefits and the risks.’(Expert within patient communication, shared decision-making, health literacy and cancer screening)*	*’I think it is important to know when deciding to say yes or no. Facts and statistics. What do I have to measure myself against. How many will get a bad answer.’ (Woman, 35 –69 years)* *’I also think it is really important, it gives a good picture of how many you are able to save from getting cancer. So, for me it would be crucial.’ (Woman, 25 –35 years)* *’No, I do not think numbers are needed. It doesn’t matter for my decision.’ (Woman, 35 –69 years)*
**Option of non-participation**	*’I think it should be presented as one of the options, and you know for the option of screening you also get several outcomes, and for the option not screening, you should also present information. I think it should be equal. And neutral. I wouldn’t say that somebody will be angry at you if you don’t screen, and you get cancer, but you could present it like equal, neutral outcome.’ (Expert within informed decision-making, health literacy and cancer screening)*	*’It is always important that the option is available for you to say no thank you. We all need to have that option; nothing in the world is forced. […] So, of course, you should have the option to say no’. (Woman, 35 –69 years)*

**Table 4:** Similarities and differences between perspectives of the experts and the citizens.

Theme	International scientific experts	Danish citizens
Disease and symptoms	Knowledge about disease and symptoms is important to engage in informed decision-making.	Several citizens mentioned knowledge about disease and symptoms as important. Some citizens felt it was not essential for their decision-making.
Screening composition	Knowledge about the screening composition was described as essential. This includes both the screening test and follow-up test.	Knowledge about the screening composition was essential for the citizens. Knowledge about potential outcomes was important for some citizens and for some citizens, knowledge about the target population was considered crucial.
Benefits and harms	Knowledge about both benefits and harms of screening was important. Detailed knowledge about the harms might not be necessary.	Knowledge about the benefits of screening was mentioned as crucial for the citizens. A minority of the citizens mentioned information about harms as crucial and essential.
Numeric information	Numeric information about the screening test was described as essential. It should be general numeric information and not too detailed.	Most of the citizens described numeric information as a useful way to make a ‘fact-based’ decision about participation. Some citizens felt that numeric information was not relevant.
Option of non-participation	Knowledge about the possible options was felt as essential. It was also described as essential to know the potential consequences of non-participation.	Most of the citizens mentioned that it was essential to have knowledge about the option of non-participation, and that they have the option to participate later if the first decision was non-participation.

## Appendix A. – Interview guides

**Table. A1:** Interview guide with experts – first interview.

Research question	Theme	Interview question
What is knowledge about cancer screening for scientific experts/researchers	Relevant knowledge for scientific experts/researchers	Thinking as broadly as possible, what is relevant knowledge about cancer screening for you as scientific expert/researcher? Why do you mention this knowledge as relevant?
What is knowledge about cancer screening for citizens	Relevant knowledge for citizens	Briefing: Now we want you to focus on what is relevant about cancer screening for the citizens. Thinking as broadly as possible, what is relevant for citizens to know about cancer screening? Can you explain more about what you think relevant knowledge about cancer screening is for the citizens? Why is the knowledge you mention relevant for the citizens to know about cancer screening? Why/why not is relevant knowledge about cancer screening for citizens different than relevant knowledge about cancer screening for you as experts? **Case** If a resident asks you about cancer screening, what would you tell the citizen? And why?
What do citizens need to know in order to make an informed choice about participation in cancer screening?	Relevant knowledge and informed choice	Briefing: Still with a focus on the citizens, we want you to focus on relevant knowledge in relation to informed decision making. What is relevant knowledge about cancer screening for citizens in order to make an informed choice about participation or non-participation? What does it mean for the citizens to have this knowledge about cancer screening in order to make an informed choice about participation/non-participation? Why does this knowledge matter in making an informed choice? What importance does this knowledge have for the participation or non-participation in cancer screening? Can you give a justification for why the relevant knowledge you mention is important in the decision-making process? Do you think relevant knowledge is different within different screening programmes? Which relevant knowledge is not necessary for citizens to have in order to make an informed choice? Should the citizens be informed about the option of non-participation? Case If a resident is going to decide whether he/she should participate in cancer screening and he/she asks you for information about cancer screening, what would you tell him/her? And why?
What is adequate knowledge?	Adequate knowledge	How do you define adequate knowledge in order to make an informed choice? Exactly knowledge do you think citizens need to have in order to make an informed choice about participation in cancer screening? What specific topics do citizens need to have knowledge about in order to make an informed choice about participation or non-participation? What specific knowledge do the citizens need to have in order to make an informed choice? Why is it/why is it not important to have specific knowledge? If citizens in general have difficulty understanding risks, probabilities and weighing up pros and cons, what specific knowledge do citizens need to have in order to make an informed choice? What importance does adequate knowledge have for the citiens’ informed decision making process?

**Table. A2:** Interview guide with experts – Second interview.

Research question	Theme	Interview question
What do the experts think about the differences between citizens’ perspectives and experts’ perspectives?	Different perspectives between citizens and experts	What do you think about the differences between the citizens’ perspectives and your perspectives? Why do you think there are these differences? Why do you think it is relevant for the citizens to have knowledge about over diagnosis, over treatment, false positives and false negatives in order to make an informed decision when the citizens do not think this is relevant for their decision? Is it still important for you? If yes - to what extent/level should the citizens have knowledge about this? The citizens do not mention that it is important for them to know that screening is a repetitive process. Do you still think it is important? If yes, why do you think it is important? From the citizens’ perspectives, it is important to have numeric knowledge about the benefits of screening, such as the effect of screening, how many lives the screening test saves and how likely the test is to find the disease. The citizens’ do not mention the potential risks of screening when they talk about screening. Do you still think it is important that the citizens have numeric knowledge about the risks? What do you mean when you say numeric knowledge about the risks? Can you maybe give an example? The citizens do not think it is important to know that screening is for healthy people. Further, they don’t think it is important to know that screening has an end-date when they make the decision at the first invitation. Instead, this is important when the end date approaches. What do you think about this differences? Do you still think the information is important? The citizens think that it is important to know that the non-participation option exists. At the same time, most of them say that it is implicit because the invitation to screening is only an ‘offer’. What they think is more important is to know that they can participate later if they say no the first time. Most of you mentioned that the citizens should have information about non-participation but is it important to measure whether the citizens have knowledge about the non-participation option when we measure their level of relevant knowledge?- Is it important to measure whether the citizens have knowledge about the option ‘non-participation’ or do they need to have knowledge about the consequences of non-participation? At the first workshop you focused on the *’gist of screening’* and *’core knowledge’ -* what do you mean when you say *’gist of screening’* or *’core knowledge?* Is *’the gist’* the same as the aim of screening or does *’the gist’* include more details? At the first workshop you also focused on the benefits and risks of cancer screening - but what exactly do you mean when you say benefits and risks?
What is relevant knowledge?	Consensus about relevant and adequate knowledge of cancer screening	Based on the differences between your perspectives and the citizens’ perspectives - what do you think is sufficiently important for the citizens to have knowledge about? Why do you mention these elements? To what extent should the citizens have knowledge about this? Are these elements important in such an extent that these should be put in a scale to measure knowledge? Should we only include the items/elements where the experts and the citizens agree? Are these items sufficiently important to put in a scale and overriding the citizens’ views? Do we need to put these items/elements in a scale even though the citizens don’t think it is important? To make a summary before we go to last question, which elements do you think are sufficiently important to be put in a scale for the measurement of knowledge of cancer screening?
How do we measure relevant knowledge about cancer screening?	Measurement of adequate knowledge about cancer screening	Would it still be possible to measure the citizens’ knowledge about colorectal, cervical and breast cancer screening with a generic scale? You point out that it is important that the citizens have numeric knowledge about benefits and harms. This numeric knowledge will be condition specific. Do you still think it is possible to develop a generic scale or a cross-condition specific scale, which will override the citizens’ views?

**Table. A3:** Interview guide with Danish citizens.

Research question	Interview question	Additional question
Opening question	Do you remember the last time you received the invitation for cancer screening?	What did you think when you received the invitation? Why did you think as you did? Did anything surprise you? Did you feel that you received enough and the right information when you had to decide whether you wanted to participate in screening? Was there too much or too little information? How was the format?
Where the citizens get their information from	Where do you get your information about cancer screening from?	Why do you prefer to get your information from there? Which information do you pay the most attention to? What is most decisive for whether you participate in screening or not? When is it most optimal to receive the information? Before you are invited or with the invitation?
Whether the citizens have the knowledge they need	What do you immediately think is the most important information to receive from the health authorities when you get an invitation to screening?	Why is that information important to you? Why is that information important to have when you have to decide whether to participate or not in cancer screening? What is the best way to receive the information you find important?
The citizens perspectives of what constitutes relevant knowledge about cancer screening (the perspectives from the citizens)	Case: Now, we will fill out an information leaflet together with information that you think is most important. I will read out some different possible pieces of information, and you will need to decide whether you think it is important to include. Examples on the information from the Danish information material were used. Information about the disease itself, such as symptoms of the disease and the risk of developing the disease during your lifetime. Information that you only have to participate in screening, if you do not have any symptoms. If you have symptoms you should see a doctor. Information about how you participate in screening. How the screening test will be conducted. The possible outcomes of a screening test, and what will happen with the different outcomes. Numbers which show the risk of getting the disease or dying from it, if you participate in screening compared to if you do not participate in screening. Information about the harms of screening, such as false positive and false negative results, over diagnosis and over treatment. Information that screening is limited to a specific age group, and an explanation of why. Information that you can choose not to participate in cancer screening.	Why do you think that? Why do you not think that? Can you try to explain more about why you think that information is important for you? Can you try to explain more? What are your thoughts on screening stopping at a certain age?

## Abbreviations:

COSMIN: COnsensus-based Standards for the selection of health Measurement INstruments
FIT: Faecal Immunochemical Testing
HPV: Human Papillomavirus
FGI: Focus group interview
HL: Health literacy
PROM: Patient-reported outcome measures
OECD: Organisation for Economic Co-operation and Development

## References

[1] Marteau TM, Dormandy E, Michie S. A measure of informed choice. Health Expect 2001;4(2):99–108. 11359540 10.1046/j.1369-6513.2001.00140.x PMC5060053

[2] Jepson RG, Hewison J, Thompson A, Weller D. Patient perspectives on information and choice in cancer screening: a qualitative study in the UK. Soc Sci Med 2007;65 (5):890–9. 17507131 10.1016/j.socscimed.2007.04.009

[3] Kirkegaard P, Mortensen GL, Mortensen SL, Larsen MB, Gabel P, Andersen B. Making decisions about colorectal cancer screening. A qualitative study among citizens with lower educational attainment. Eur J Public Health 2016;26(1):176–81. 26541860 10.1093/eurpub/ckv207

[4] Reder M, Berens E-M, Spallek J, Kolip P. Development of the Informed Choice in Mammography Screening Questionnaire (IMQ): factor structure, reliability, and validity. BMC Psychol 2019;7(1). 10.1186/s40359-019-0291-2 PMC6423759 30890190

[5] Stokholm RN, Stenholt L, Lauridsen HH, Edwards A, Andersen B, Larsen MB. The validity of instruments to measure knowledge in population-based cancer screening targeting individuals at average risk - a systematic review. Prev Med 2024;182:107940. 38513839 10.1016/j.ypmed.2024.107940

[6] de Vet HCW, Terwee CB, Mokkink LB, Knol DL. Measurement in Medicine: A Practical Guide. Cambridge University Press; 2011.

[7] Malterud K, Siersma VD, Guassora AD. Sample size in qualitative interview studies: guided by information power. Qual Health Res 2016;26(13):1753–60. 26613970 10.1177/1049732315617444

[8] Sorensen K, van den Broucke S, Pelikan JM, Fullam J, Doyle G, Slonska Z, Measuring health literacy in populations: illuminating the design and development process of the European Health Literacy Survey Questionnaire (HLS-EU-Q). BMC Public Health 2013;13(1):948. 24112855 10.1186/1471-2458-13-948 PMC4016258

[9] Sorensen K, Pelikan JM, Roethlin F, Ganahl K, Slonska Z, Doyle G, Health literacy in Europe: comparative results of the European health literacy survey (HLS-EU). Eur J Public Health 2015;25(6):1053–8. 25843827 10.1093/eurpub/ckv043 PMC4668324

[10] Horshauge PM, Gabel P, Larsen MB, Kirkegaard P, Edwards A, Andersen B. The association between health literacy and colorectal cancer screening uptake in a publicly funded screening program in Denmark: cross-sectional study. Prev Med Rep 2020;19:101132. 32551215 10.1016/j.pmedr.2020.101132 PMC7287294

[11] Gabel P, Larsen MB, Edwards A, Kirkegaard P, Andersen B. Knowledge, attitudes, and worries among different health literacy groups before receiving first invitation to colorectal cancer screening: cross-sectional study. Prev Med Rep 2019;14: 100876. 31080706 10.1016/j.pmedr.2019.100876 PMC6506556

[12] Braun V, Clarke V. Using thematic analysis in psychology. Qual Res Psychol 2006;3(2):77–101.

[13] Shenton AK. Strategies for ensuring trustworthiness in qualitative research projects. Educ Inf 2004;22(2):63–75.

[14] Woudstra AJ, Timmermans DRM, Uiters E, Dekker E, Smets EMA, Fransen MP. Health literacy skills for informed decision making in colorectal cancer screening: perceptions of screening invitees and experts. Health Expect 2018;21(3):636–46. 29266661 10.1111/hex.12658 PMC5980534

[15] International Patient Decision Aid Standards (IPDAS) Collaboration, 〈http://ipdas.ohri.ca/〉; 2024. [Accessed 02 September 2024].

[16] Stacey D, Volk RJ. The International Patient Decision Aid Standards (IPDAS) Collaboration: Evidence Update 2.0. Med Decis Mak 2021;41(7):729–33. 10.1177/0272989X211035681 PMC8474333 34416841

[17] Fransen MP, Damman OC, Bas S, Uiters E, Timmermans DRM. Decision-making in breast cancer screening: a qualitative exploration of the match between women’s beliefs and screening information in the Netherlands. Patient Educ Couns 2024; 122:108155. 38325207 10.1016/j.pec.2024.108155

[18] Hersch J, Jansen J, Barratt A, Irwig L, Houssami N, Howard K, Women’s views on overdiagnosis in breast cancer screening: a qualitative study. Bmj 2013; 346:f158. 23344309 10.1136/bmj.f158 PMC3552499

[19] Pappadis MR, Volk RJ, Krishnan S, Weller SC, Jaramillo E, Hoover DS, Perceptions of overdetection of breast cancer among women 70 years of age and older in the USA: a mixed-methods analysis. BMJ Open 2018;8(6):e022138. 10.1136/bmjopen-2018-022138 PMC6009543 29903800

[20] Housten AJ, Lowenstein LM, Hoffman A, Jacobs LE, Zirari Z, Hoover DS, A review of the presentation of overdiagnosis in cancer screening patient decision aids. MDM Policy Pract 2019;4(2). 2381468319881447–2381468319881447. 10.1177/2381468319881447 PMC8855414 35187246

[21] Kolthoff SK, Hestbech MS, Jørgensen KJ, Brodersen J. Do invitations for cervical screening provide sufficient information to enable informed choice? A cross-sectional study of invitations for publicly funded cervical screening. J R Soc Med 2016;109(7):274–81. 27118696 10.1177/0141076816643324 PMC4940995

[22] Mathieu E, Barratt A, Davey HM, McGeechan K, Howard K, Houssami N. Informed choice in mammography screening: a randomized trial of a decision aid for 70-year-old women. Arch Intern Med 2007;167(19):2039–46. 17954796 10.1001/archinte.167.19.2039

[23] Mathieu E, Barratt AL, McGeechan K, Davey HM, Howard K, Houssami N. Helping women make choices about mammography screening: an online randomized trial of a decision aid for 40-year-old women. Patient Educ Couns 2010;81(1):63–72. 20149953 10.1016/j.pec.2010.01.001

[24] Hersch J, Nickel B, Ghanouni A, Jansen J, McCaffery K. Improving communication about cancer screening: moving towards informed decision making. Public Health Res Pract 2017;27:e2731728. 10.17061/phrp2731728). 28765861

[25] Seaman K, Dzidic PL, Castell E, Saunders C, Breen LJ. A systematic review of women’s knowledge of screening mammography. Breast 2018;42:81–93. 30199761 10.1016/j.breast.2018.08.102

[26] Mullen PD, Allen JD, Glanz K, Fernandez ME, Bowen DJ, Pruitt SL, Measures used in studies of informed decision making about cancer screening: a systematic review. Ann Behav Med 2006;32(3):188–201. 17107291 10.1207/s15324796abm3203_4

